# Comprehensive analysis of RNA m6A methylation in pressure overload-induced cardiac hypertrophy

**DOI:** 10.1186/s12864-022-08833-w

**Published:** 2022-08-11

**Authors:** Weidong Li, Chenxv Xing, Limeng Bao, Shengna Han, Tianxia Luo, Zhiju Wang, Hongkun Fan

**Affiliations:** 1 Department of Physiology and Neurobiology, School of Basic Medical Sciences, Zhengzhou University, No.100 Kexuedadao Road, Zhengzhou, 450000 China; 2 Department of Pharmacology, School of Basic Medical Sciences, Zhengzhou University, No.100 Kexuedadao Road, Zhengzhou, 450000 China; 3 Department of Physiology, School of Medicine, Henan University of Chinese Medicine, No.156 Jinshui Road, Zhengzhou, 450052 China

**Keywords:** Cardiac hypertrophy, m^6^A transcriptome, MeRIP-seq

## Abstract

**Aim:**

To analyze and compare the mRNA N^6^-methyladenosine modifications in transverse aortic constriction induced mice hearts and normal mice hearts.

**Materials and methods:**

Colorimetric quantification was used to probe the changes in m^6^A modifications in the total RNA. The expression of m^6^A-related enzymes was analyzed via qRT-PCR and western blotting. RNA-seq and MeRIP-seq were performed to identify genes with differences in m^6^A modifications or expression in the transcriptome profile.

**Results:**

Compared with the control group, the TAC group exhibited higher m^6^A methylation levels. FTO and WTAP were downregulated after TAC, while METTL3 was significantly downregulated at the protein level. MeRIP-seq revealed that 1179 m^6^A peaks were upmethylated and 733 m^6^A peaks were downmethylated, and biological analysis of these genes exhibited a strong relationship with heart function.

**Conclusion:**

Our findings provide novel information regarding m^6^A modification and gene expression changes in cardiac hypertrophy, which may be fundamental for further research.

**Supplementary Information:**

The online version contains supplementary material available at 10.1186/s12864-022-08833-w.

## Introduction

Cardiac hypertrophy is an adaptive response to myocardial contractility and cardiac function [1]. Cardiac tissues exhibit plasticity and can respond to changes in the external environment [2]. Cardiac hypertrophy is characterized by myocardial thickening, for reasons unclear, as well as increased myocardial cell volume, increased protein synthesis, sarcomere remodeling, and myocardial fibrosis. The continuous development of cardiac hypertrophy, induced by various mechanical stimuli and neurohumoral factors, may eventually lead to ventricular dilation, heart failure, malignant arrhythmias, or sudden death [3, 4]. However, there has been no significant breakthrough in the research on blocking and reversal of myocardial remodeling.

The N^6^-methyladenosine (m^6^A) modification is a post-transcriptional modification that is widely present in all regions of the mRNA [5]. Although early studies have confirmed this modification as the most common and abundant type of mRNA modification, its function is not clear [6]. Jia et al*.* (2011) [7] found that the fat mass and obesity-associated (FTO) protein is related to the reversible modification of m^6^A in the mRNA. Using siRNA to induce the degradation of FTO mRNA results in an increase in the amount of m^6^A in the mRNA, while the construction of specific vectors to express *FTO* leads to decreased m^6^A levels in the cells. Subsequently, experimental data have increasingly shown that the RNA m^6^A methylation requires the participation of three types of molecules: writers, erasers, and readers [8]. Methyltransferase-like 3 (METTL3) and methyltransferase-like 14 (METTL14) are the most common writers, and are primarily responsible for the addition of methyl groups to the sixth N atom of specific nucleotide bases in the mRNA, whereas FTO and AlkB homolog 5 (ALKBH5) act as erasers for demethylation to remove m^6^A modifications [9–11]. Readers are responsible for recognizing and exerting m^6^A modifications in the mRNAs [12–14]. For example, YTH domain-containing protein 1 recognizes m^6^A modifications in the mRNA and promotes mRNA export, translation, and alternative splicing [15, 16]. Currently, studies are being conducted on the regulation of m^6^A RNA methylation in various cardiovascular diseases. However, post-transcriptional regulation of the mRNA, which can affect the expression of key proteins and cardiac function in pathological cardiac hypertrophy and heart failure, remains in the initial exploration phase.

In this study, to further explore the relationship between m^6^A modification and myocardial hypertrophy and to study the role of m^6^A modification in the development of myocardial hypertrophy in mice, we obtained the whole-transcriptome profiles of altered m^6^A modifications after hypertrophy using RNA-seq and MeRIP-seq techniques on normal and hypertrophic mice heart tissues, and investigated the biological functions of these differential m^6^A modifications and gene expressions genes. We believe that this research will be useful for further therapy in cardiac hypertrophy.

## Materials and methods

### Animals and tissue collection

In this study, male C57BL/6 J mice (aged six weeks) were used and randomly assigned to transverse aortic constriction (TAC) or control groups. Prior to surgery, all mice were housed under a 12 h light/dark cycle with free access to food and water for at least seven days. Four weeks after TAC was performed by ligating the aortic arch between the brachiocephalic trunk and left common carotid artery through the placement of a 27-gauge needle, mice in both groups were sacrificed under isoflurane anesthesia. Thereafter, the heart tissues of both groups were collected and stored at -80 °C until further experimentation. All experimental procedures were approved by the animal ethics committee at the University of Zhengzhou, China (SCXK-2017–0001).

### Cardiac (echocardiography) function

Mice in both groups were anesthetized with 1.5% isoflurane and M-mode echocardiograms were used to detect cardiac function. Ejection fraction (EF%), fractional shortening (FS%), internal dimension at end-diastole (LVID.d) and at end-systolic (LVID.s), left ventricular posterior wall thickness at end-systolic (LVPW.s) and at end-diastole (LVPW.d), interventricular septal thickness at end-systolic (IVS.s) and at end-diastole (IVS.d) were measured.

### Histology analysis

The heart weight normalized to body weight (HW/BW) and lung weight normalized to body weight (LW/BW) were measured after the mice were killed. For histopathological studies, the paraffin-embedded heart tissues were sectioned at a thickness of 5 µm. Hematoxylin & eosin (H&E) and Masson’s trichrome staining were performed on histological sections obtained from the paraffin-embedded hearts. Wheat germ agglutinin (WGA) staining was used to analyze cardiac hypertrophy.

### RNA preparation

Total RNA was extracted from the heart tissues of mice in both groups using TRIzol reagent (Invitrogen, Waltham, MA, USA), according to the manufacturer’s instructions. The quality and quantity of the total RNA was evaluated by using NanoDrop-2000 (Thermo Fisher Scientific, Waltham, MA, USA).

### Quantification of m^6^A in total RNA

Total RNA from six samples per group was used for the quantification of m^6^A modifications using the colorimetric quantification method (P-9005, EpiGentek, Farmingdale, NY, USA). Briefly, a single-point positive control was used, as recommended by the manufacturer. Total RNA (200 ng), negative control, and diluted positive control were added to 96-well plates and a binding solution was used to bind RNA to the 96-well plates. The binding solution from each well was removed after 90 min and each well was washed three times with diluted wash buffer. Next, the capture antibody, detection antibody, and enhancer solution were added to capture m^6^A RNA and each well was washed with diluted wash buffer after each step. Finally, the developer solution and stop solution were added to each well to stop the enzyme reaction. The absorbance of each well was read using a microplate reader at 450 nm, and the percentage of m^6^A in total RNA was calculated.

### Quantitative real-time polymerase chain reaction (qRT-PCR)

mRNA expression levels of m^6^A-related genes *METTL3*, *WTAP*, *ALKBH5*, *FTO*, and *METTL14* were analyzed in the TAC group and sham control using qRT-PCR. In brief, total RNA was extracted from both groups as previously described, and cDNA was obtained via reverse transcription using the PrimeScript™ RT reagent Kit with gDNA Eraser (Perfect Real Time, TakaraBio, Japan). RT-PCR was performed using the TB Green Master Mix (Tli RNaseH Plus, TakaraBio). All procedures were performed in accordance with the manufacturer’s instructions. The primer sequences used are listed in Table 1.

**Table 1:** Primers used for qRT-PCR

Genes	Primer types	Primer Sequences (5’-3’)
*METTL3*	Forward	CGCTGCCTCCGATGTTGATCTG
*METTL3*	Reverse	CTGACTGACCTTCTTGCTCTGCTG
*METTL14*	Forward	TGCAGCACCTCGGTCATTTA
*METTL14*	Reverse	TAACCCCACTTTCGCAAGCA
*WTAP*	Forward	GAAGGAGACACGACAGCAGTTGG
*WTAP*	Reverse	GCTTGTGACCTCTGCCTGATCTAC
*FTO*	Forward	ATGAAGACGCTGTGCCACTGTG
*FTO*	Reverse	CACGTTGTAGGCTGCTCTGCTC
*ALKBH5*	Forward	GCAAGGTGAAGAGCGGCATCC
*ALKBH5*	Reverse	GTCCACCGTGTGCTCGTTGTAC
*GAPDH*	Forward	GGTTGTCTCCTGCGACTTCA
*GAPDH*	Reverse	TGGTCCAGGGTTTCTTACTCC

### Western blotting

Proteins were extracted from the heart tissues using RIPA lysis buffer (Solarbio, Beijing, China) containing 1 mmol/L phenylmethylsulfonyl fluoride (Solarbio) for western blotting analysis. Briefly, approximately 50 μg protein was subjected to sodium dodecyl sulfate–polyacrylamide gel electrophoresis and transferred to a polyvinylidene membrane (Millipore, Burlington, MA, USA), which was then incubated with the primary antibodies. Then, the membranes were incubated with horseradish peroxidase (HRP)-conjugated goat anti-mouse IgG or HRP-conjugated goat anti-rabbit IgG after washing with tris-buffered saline containing 0.1% Tween 20. Glyceraldehyde 3-phosphate dehydrogenase was used as the internal control. The antibodies used in this study were METTL3 (1:2000; 15,073–1-AP, ProteinTech, Rosemont, IL, USA), ALKBH5 (1:10,000; 16,837–1-AP, ProteinTech), WTAP (1:10,000; 60,188–1-Ig, ProteinTech), FTO (1:2000; ab280081, Abcam, Cambridge, UK), BMP4 (1:1000; ab39973, Abcam, Cambridge, UK), and GAPDH (1:20,000; 60,004–1-Ig, ProteinTech).

### MeRIP-seq and RNA-seq

After four weeks of transverse aortic constriction surgery, three biological replicates were performed for sham and TAC group, we randomly selected three samples from each group for sequence. The m^6^A-IP-Seq and RNA-seq services were provided by CloudSeq, Inc., Shanghai, China. After four weeks of TAC, total RNA was extracted from the heart tissues of mice in both groups, and 50 µg of mouse total RNA (TAC and control) was used for MeRIP-seq. Briefly, before RNA was immunoprecipitated with the m^6^A antibody, total RNA was randomly fragmented to obtain the fragments of 200 nt. Protein A/G beads were used to combine the m^6^A antibody through rotation for 1 h at room temperature. Afterward, the RNA fragments were added to the immunocapture solution and rotated for 4 h to ensure proper mixing. The beads were then resuspended with Elution Buffer to release the RNA.

For RNA-seq, rRNAs should be removed from total RNA using NEBNext rRNA Depletion Kit (New England Biolabs, Inc., Ipswich, MA, USA). RNA libraries of m6A-IP-Seq and RNA-seq were constructed using the NEBNext® Ultra II Directional RNA Library Prep Kit (New England Biolabs Inc.), and the libraries were qualified using an Agilent 2100 BioAnalyzer system (Agilent Technologies, Inc., Santa Clara, CA, USA) and sequenced on a NovaSeq platform (Illumina, Inc., San Diego, CA, USA).

Briefly, raw data were obtained after quality controlled with Q30. Cutadapt software v1.9.3 was used to remove low-quality reads, and Hisat2 software v2.0.4 was used to align the clean reads to the reference genome [17]. To identify m^6^A methylated regions on the mRNA and differentially methylated sites between the two groups, MACS and diffReps software were used [18, 19]. Altered m^6^A peaks with fold change > 2 and *p*-value undergo false discovery rate < 0.01 (*p*-value cutoff of < 0.0001 were default selection criteria of diffReps software, then undergo false discovery rate correction) were selected for gene ontology and pathway enrichment analyses [20–23].

The differentially methylated and expression genes were divided into two groups (upregulation or downregulation) and subjected to gene ontology analysis and pathway enrichment analysis. The background gene list used in this analysis were detected genes in RNA-seq, and genes with an average of less than 10 reads per sample were omitted. The gene ontology analysis was performed using (www.geneontology.org, current release 2022–03) and pathway enrichment analysis was performed using clusterProfiler R package (v4.2), the *p*-value denotes the significance of GO term enrichment of the genes, the Fisher *p*-value denotes the significance of the pathway correlated to the conditions. Then all *p*-value undergo FDR (Benjamini & Hochberg) correction.

### MeRIP-qPCR

In this experiment, EpiQuikTM CUT&RUN m^6^A RNA Enrichment (MeRIP) Kit (P-9018, EpiGentek, Farmingdale, NY, USA) was used to enrich an RNA fragment containing m^6^A. After 4/8 weeks of TAC, the heart tissues were collected and stored at -80 °C. Total RNA was obtained as previously described, an optimal input RNA amount is 10 ug per reaction. All procedures were performed in accordance with the manufacturer’s instructions. Briefly, adding m6A antibody, affinity beads and immuno capture buffer to PCR tubes. After rotate the tubes for 90 min, add nuclear digestion enhancer and cleavage enzyme mix reagents for cleavage under targets. Proteinase K and RNA binding beads were used for RNA purification. At last, resuspend the beads in 13 ul of elution buffer and transfer 13ul to a new PCR tube. The enriched RNA was used for qRT-PCR. The primer sequences used are listed in Table 2.

**Table 2:** Primers used for MeRIP-qPCR

Genes	Primer types	Primer Sequences (5’-3’)
*KCNN2*	Forward	CGCGTTTATTTTTGGCGCA
*KCNN2*	Reverse	GGAGAGCACTGGTGGACG
*BMP4-1*	Forward	CCCACTGAACTGAGTGCCAT
*BMP4-1*	Reverse	CATCCACACCCCTCTACCAC
*BMP4-2*	Forward	GCCGGGGCCATACCTTGA
*BMP4-2*	Reverse	GGCGACGGCAGTTCTTATTC

### Statistical analyses

Data are expressed as the means ± standard deviation (SD). The unpaired Student’s *t*-test was used to determine statistical significance for experiments with two groups, and a *p*-value < 0.05 was considered statistically significant (*p < 0.05, **p < 0.01).

## Results

### Cardiac hypertrophy in mice four weeks post TAC

**Fig. 1:**
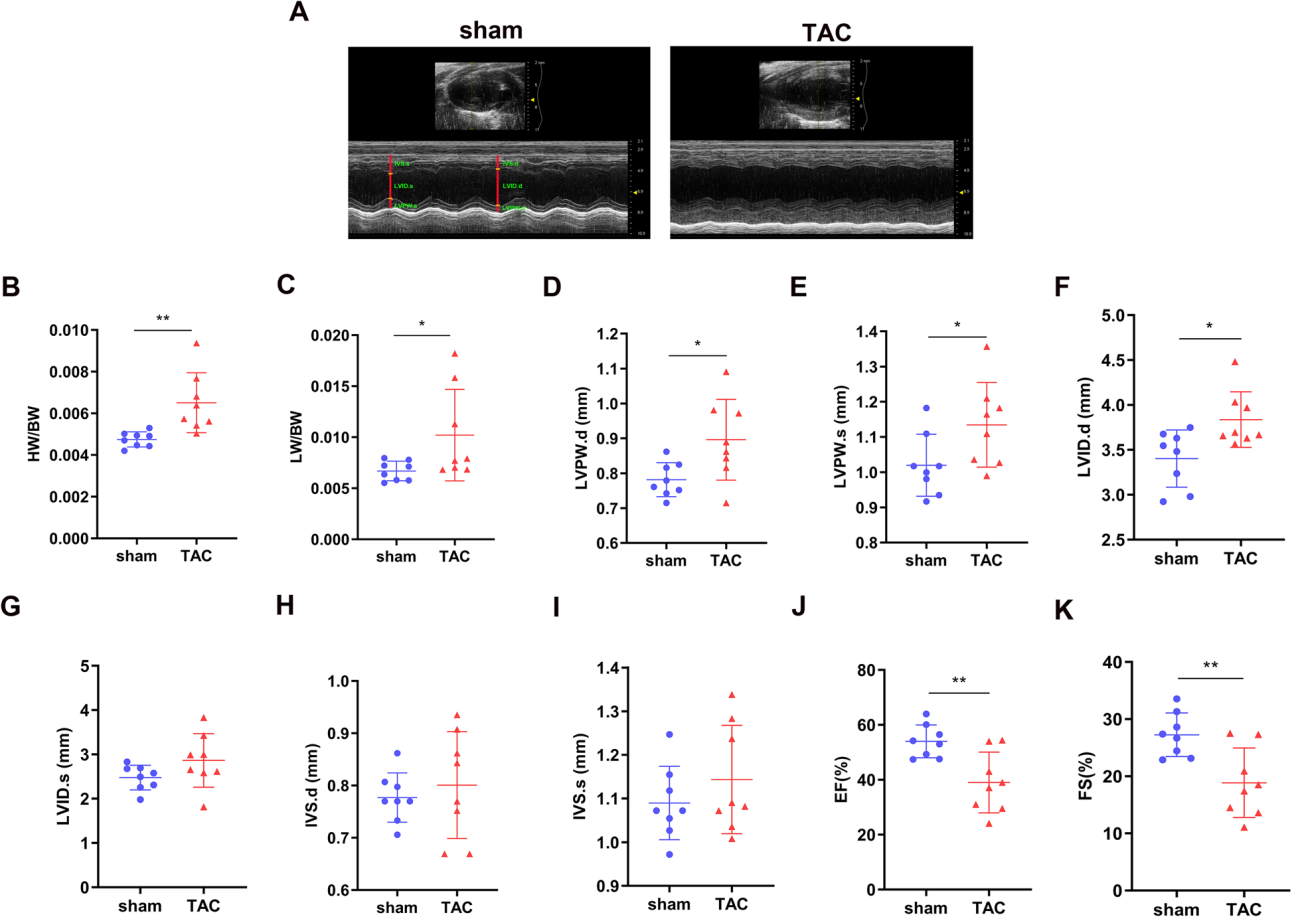
Ventricular hypertrophy and reduced cardiac function were observed in mice after four weeks of transverse aortic constriction. **A** Representative image of mouse hearts in both groups; **B**–**C** Gravimetric analysis of heart weight normalized to body weight (HW/BW) and lung weight normalized to body weight (LW/BW) in both groups (*n* = 8 each); **D**–**I** Measurements of left ventricular posterior wall thickness at end-diastole (LVPW.d), left ventricular posterior wall thickness at end-systolic (LVPW.s), internal dimension at end-diastole (LVID.d), internal dimension at end-systolic (LVID.s), interventricular septal thickness at end-diastole (IVS.d), and interventricular septal thickness at end-systolic (IVS.s) in both groups (*n* = 8 each), respectively; **J**–**K** Echocardiographic ejection fraction (EF) and fractional shortening (FS) measurements in both groups (*n* = 8 each). The results are expressed as the means ± SD (*p < 0.05, **p < 0.01, compared to the Sham group)

M-mode echocardiograms were used to detect the ventricular structure and function of the mice in both groups (Fig. 1A). Compared with the sham group, HW/BW and LW/BW were significantly higher in the TAC group (Fig. 1B and C). Furthermore, compared with the sham group, the LVPW.d, LVPW.s, and LVID.d were significantly higher in the TAC group after four weeks; while the LVID.s, IVS.d, and IVS.s showed an increasing trend in the TAC group, but did not increase significantly (Fig. 1D–I). The ejection fraction reflects the ventricular ejection function; the left ventricular fractional shortening is an important indicator for evaluating cardiac contractility. Four weeks post TAC, the ejection function and cardiac contractility of mice in the TAC group were significantly lower than those of mice in the sham group (Fig. 1J–K).

**Fig. 2:**
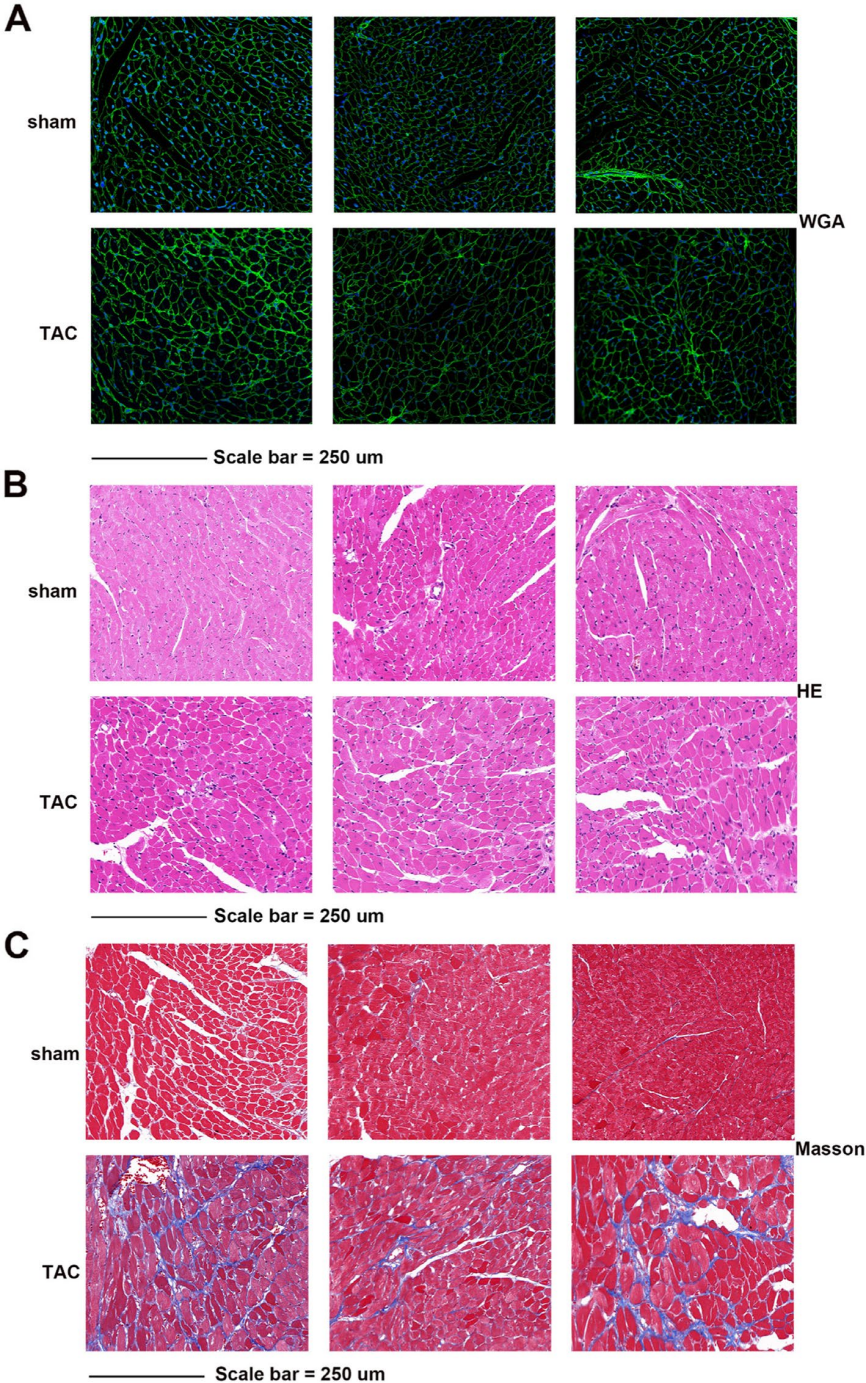
Cardiac remodeling occurred after transverse aortic constriction. **A** Representative wheat germ agglutinin-stained cardiac cross-sections from the transverse aortic constriction (TAC) and control groups. **B** Representative hematoxylin and eosin-stained cardiac cross-sections from TAC and control groups; **C** Representative results of Masson’s trichrome staining to assess fibrosis of mice heart tissues; Scale bar = 250 µm

To further verify whether the mice developed cardiac hypertrophy at four weeks post TAC, H&E, Masson’s trichrome, and WGA staining were performed on histological sections generated from paraffin-embedded mice hearts. The results showed that the cardiomyocyte morphology of mice in the TAC group was altered. Compared to the sham group, the cardiomyocytes of mice in the TAC group appeared hypertrophied (Fig. 2A and B). Myocardial fibrosis in the TAC mice was simultaneously aggravated and the collagen deposition area increased at four weeks post TAC (Fig. 2C).

### m^6^A methylation profiles in normal and hypertrophic mice hearts

**Fig. 3:**
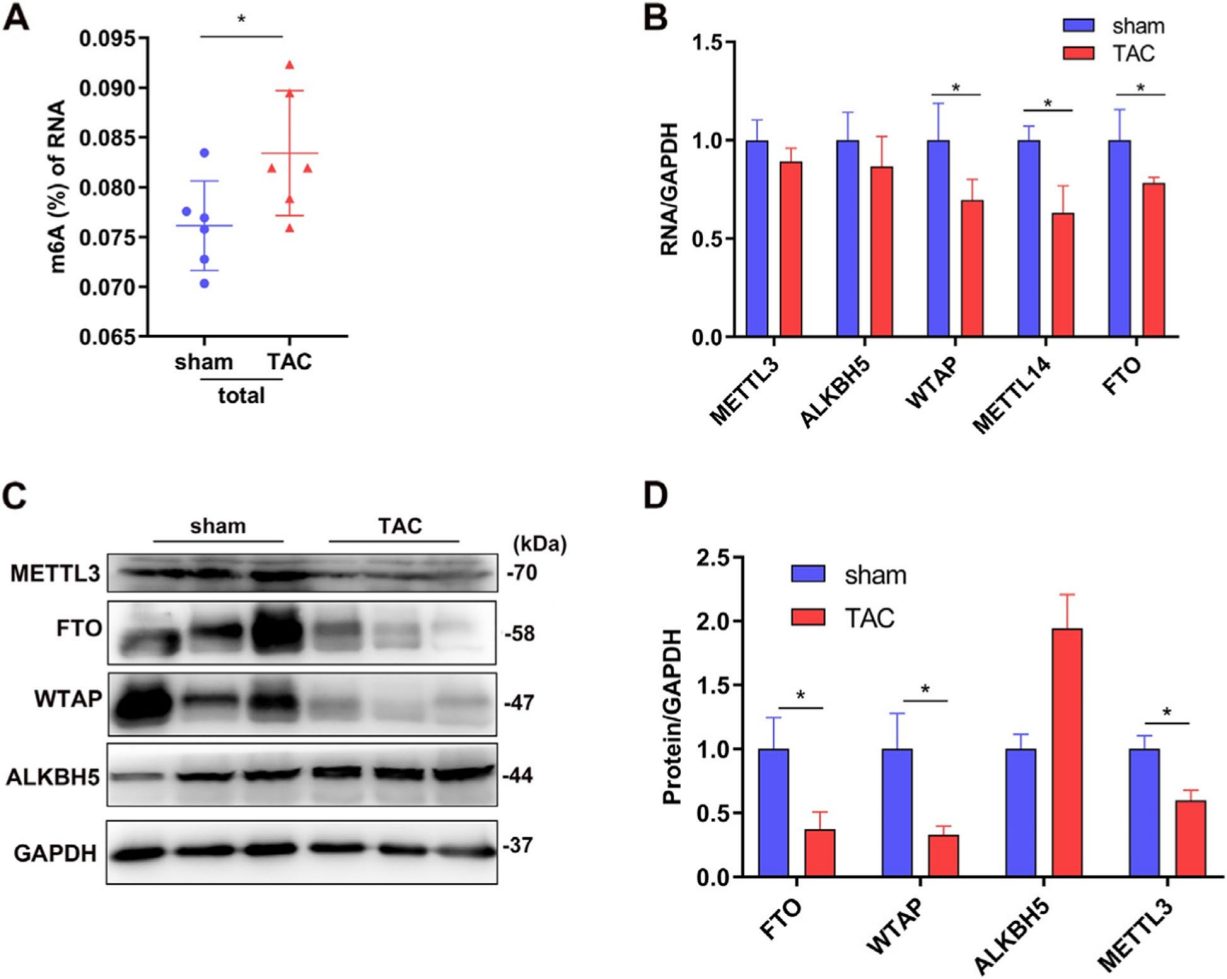
Changes in m^6^A levels after transverse aortic constriction were caused by increased methyltransferase or decreased demethyltransferases. **A** Increased m.^6^A in RNA in mice hypertrophic hearts; **B** qRT-PCR was performed to analyze mRNA levels of *METTL3*, *ALKBH5*, *WTAP*, *METTL14*, and *FTO* in both groups (*n* = 6 each); **C** Representative immunoblots; **D** Densitometry quantification of protein (*n* = 3 ~ 5 each). The results are expressed as the means ± SD (*p < 0.05, compared to the sham group)

First, we quantified m^6^A levels in the heart tissues after TAC. The results showed that m^6^A modification of total RNA was significantly higher when the mice myocardium developed hypertrophy (Fig. 3A). Based on qRT-PCR analysis, the mRNA relative expressions of five major m^6^A related enzymes, including *METTLL3*, *METTL14*, *WTAP*, *FTO* and *ALKBH5*, were compared between the sham and TAC groups. Four weeks post TAC, the mRNA levels of *METTL14* and *WTAP*, key methyltransferases responsible for m^6^A modifications, and the major demethyltransferase *FTO* were significantly lower in the TAC group compared with those of the sham group. Further, *METTL3* and *ALKBH5* were not significantly dysregulated (compared with the sham group) (Fig. 3B). To further investigate the reasons for the m^6^A modification changes in total RNA after TAC, we assessed the protein expression levels of *METTL3*, *WTAP*, *FTO*, and *ALKBH5* in the heart tissues. *FTO*, *WTAP* and *METTL3* downregulation were verified using western blotting; while the expression of demethyltransferase *ALKBH5* was upregulated, but not significantly (Fig. 3C–D).

**Fig. 4:**
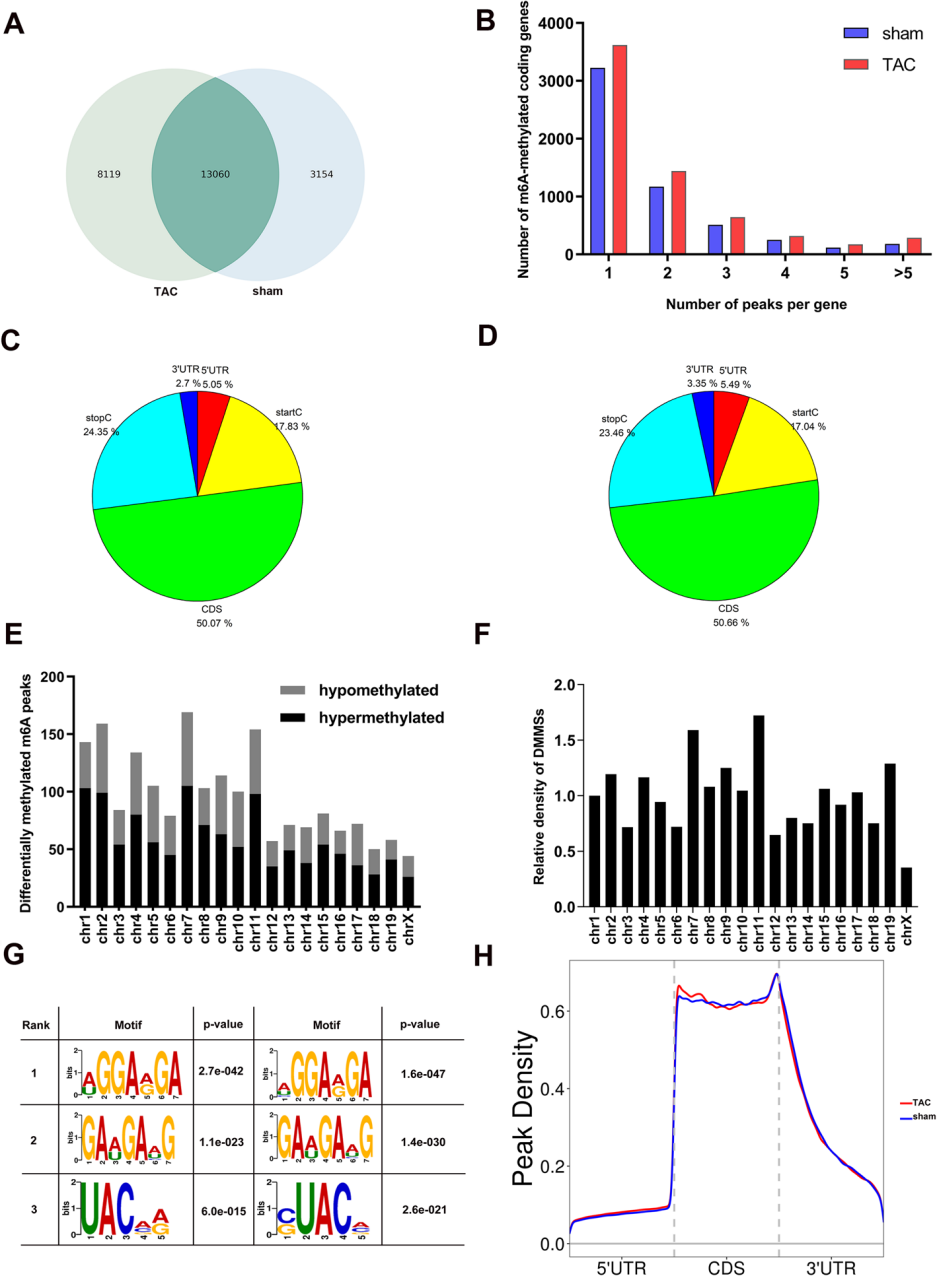
Overview of N^6^-methyladenosine methylation within mRNAs in the control and transverse aortic constriction groups. **A** Number of overlapped and non-overlapped methylation peaks in the transverse aortic constriction (TAC) and sham groups; **B** Proportion of genes harboring different numbers of m^6^A peaks in the two groups; **C** Pie charts illustrating the percentage of m^6^A peaks in five non-overlapping segments of transcripts in the sham group; **D** Pie charts illustrating the percentage of m^6^A peaks in five non-overlapping segments of transcripts in the TAC group; **E** Distribution of differentially methylated m^6^A peaks in chromosomes; **F** Relative density of differentially methylated m^6^A sites in each chromosome; **G** Top three motifs in the TAC and sham groups; **H** Metagene plots showing the preferential location of m^6^A in the mRNA

MeRIP-seq analysis of mRNA derived from mice hearts revealed 16,214 m^6^A peaks in the control group and 21,179 m^6^A peaks in the TAC group. Of these, 13,060 peaks overlapped between the control and TAC groups (Fig. 4A). All methylated N^6^-methyladenosine genes were divided into several groups according to the number of peaks per gene. Notably, most of the m^6^A-methylated coding genes in both groups contained only one or two m^6^A sites (Fig. 4B). Moreover, the number of genes in each group clearly showed that the m^6^A modification peaks in the TAC group were significantly higher than those in the sham group, which proves that the m^6^A modification level increased after TAC.

**Fig. 5:**
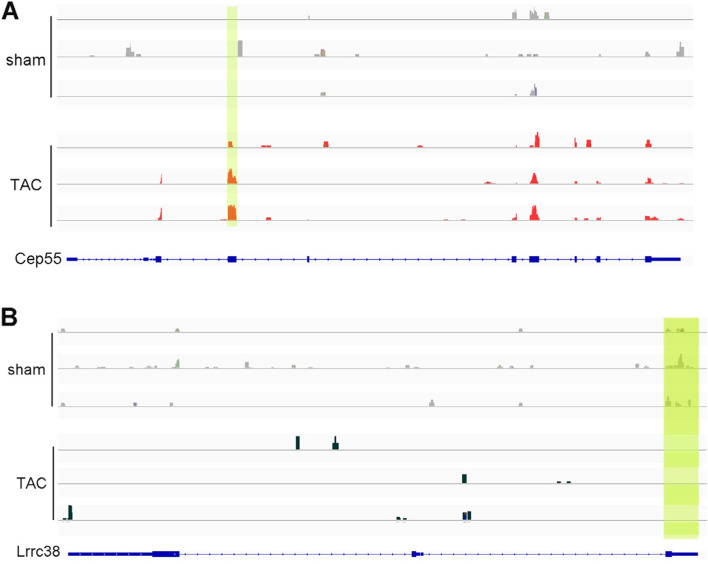
Differentially methylated m^6^A peaks in the sham and transverse aortic constriction groups. **A** Hypermethylated gene visualized in the Integrative Genomics Viewer; **B** Hypomethylated gene visualized in the Integrative Genomics Viewer

Compared with the sham group, 1179 significantly hypermethylated m^6^A peaks and 733 significantly hypomethylated m^6^A peaks were identified in the TAC group (fold change > 2 and *p*-value < 0.01). The top ten upregulated and downregulated m^6^A peaks are listed in Table 3. To analyze the distribution profiles of the m^6^A peaks within mRNAs, the peaks were categorized into five transcript segments: 5′UTR, start codon segment, coding sequence (CDS), stop codon segment, and 3′UTR [24]. Our results showed that the m^6^A sites were typically harbored in the CDS and stop codon segments (Fig. 4C–D). Furthermore, the distribution profiles of differentially methylated m^6^A peaks and relative density of differentially methylated m^6^A sites (DMMSs) in the chromosomes revealed that the dysregulated m^6^A peaks were transcribed from all chromosomes, but genes located on chr7 and chr11 most likely underwent m^6^A methylation changes (Fig. 4E and F). In this study, the first rank of m^6^A motifs were characterized “AGGAAGA” in both groups (Fig. 4G). To investigate the preferential location of m^6^A modifications in different regions of the mRNA, metagene profiles were used to visualize the differences between the two groups. The results showed that the main differences between the two groups were concentrated in the CDS region (Fig. 4H). A representation of the significantly upmethylated and downmethylated peaks is shown in Fig. 5.

**Table 3:** The top 20 differently methylated m^6^A peaks

Chromsome	txStart	txEnd	Peak length	Gene	Fold change	*P*-value	Regulation
chr11	40,707,564	40,707,717	153	Hmmr	285.9	4.6929E-09	up
chr7	80,309,392	80,309,488	96	Prc1	203.4	5.21894E-09	up
chr2	152,912,121	152,912,360	239	Mylk2	162	3.60657E-09	up
chr19	38,060,080	38,060,200	120	Cep55	160.9	3.73133E-09	up
chr9	85,844,141	85,844,580	439	Tpbg	154.2	7.1048E-13	up
chr11	78,304,045	78,304,280	235	Spag5	146.4	1.05539E-11	up
chr19	37,420,301	37,420,620	319	Kif11	145.2	2.68469E-09	up
chr3	8,993,740	8,993,820	80	Tpd52	142.9	1.91831E-11	up
chr1	134,327,257	134,327,360	103	Ppfia4	128.3	6.51387E-09	up
chr10	93,464,061	93,464,185	124	Lta4h	127.3	7.89094E-09	up
chr2	54,084,401	54,085,060	659	Rprm	186.3	1.1801E-09	down
chr17	13,227,021	13,227,097	76	Smok2a	152.1	2.92825E-09	down
chr4	143,370,101	143,370,440	339	Lrrc38	138.8	3.4094E-09	down
chr17	46,304,156	46,304,460	304	Abcc10	132.9	1.0497E-09	down
chr14	47,292,061	47,292,280	219	Socs4	132.6	3.22531E-10	down
chr10	104,185,961	104,185,992	31	Gm20765	132.4	1.26028E-09	down
chr5	114,612,781	114,612,980	199	Fam222a	123	1.41896E-11	down
chr10	122,449,061	122,449,460	399	Avpr1a	116.1	2.46993E-11	down
chr14	110,754,701	110,754,980	279	Slitrk6	114.9	3.24827E-09	down
chr15	76,715,301	76,715,580	279	Lrrc24	114.6	3.95801E-09	down

### Gene Ontology (GO) and Kyoto Encyclopedia of Genes and Genomes (KEGG) pathway analyses of differentially methylated mRNA

**Fig. 6:**
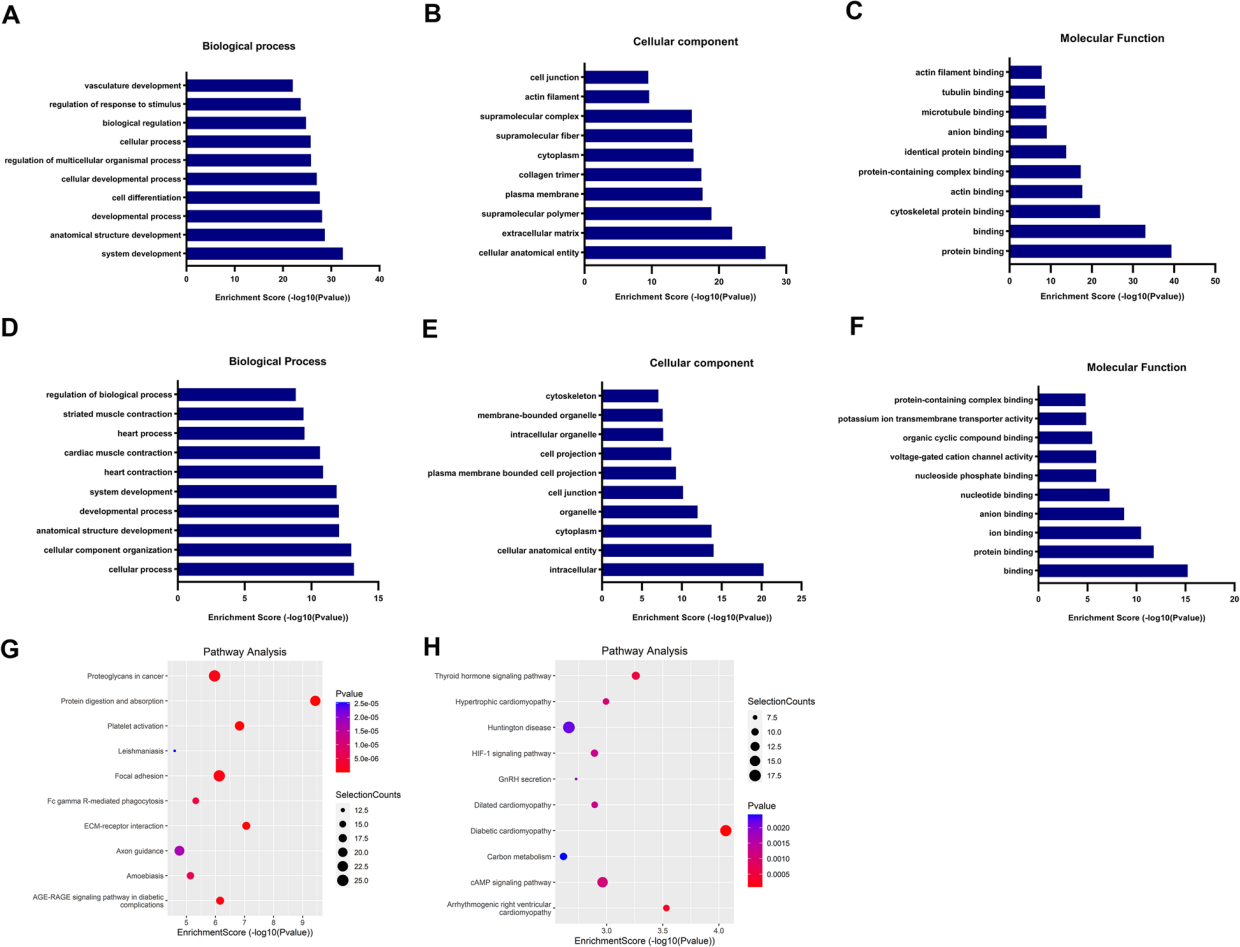
Gene ontology enrichment and pathway analysis of altered m^6^A transcripts. **A**–**C** Major and meaningful Gene Ontology (GO) terms of biological processes, cellular component, and molecular function were significantly enriched for the upmethylated transcripts; **D**–**F** Major and meaningful GO terms of biological processes, cellular component, and molecular function were significantly enriched for the downmethylated transcripts; **G** Dot plot showing top ten significant enrichment pathways for the upmethylated transcripts; **H** Dot plot showing top ten significant enrichment pathways of the downmethylated transcripts

To explore the functions of m^6^A modifications in hypertrophic mice hearts, differentially methylated m^6^A peaks were selected for GO and KEGG pathway analysis. GO analysis revealed that the upregulated peaks in the hypertrophic heart tissues were significantly associated with the system development and anatomical structure development (Fig. 6A), cellular anatomical entity and extracellular matrix (Fig. 6B), and protein binding and binding (Fig. 6C). The downmethylated peaks were significantly associated with heart processes and heart contraction (Fig. 6D), intracellular and cellular anatomical entity (Fig. 6E), and binding and protein binding (Fig. 6F).

Pathway analysis showed that the upmethylated peaks in hypertrophic heart tissues were significantly associated with focal adhesion and extracellular matrix-receptor interaction (Fig. 6G). The downmethylated peaks were significantly associated with hypertrophic cardiomyopathy and diabetic cardiomyopathy (Fig. 6H).

### Analysis of RNA-binding proteins (RBPs) of differentially methylated mRNAs

**Fig. 7:**
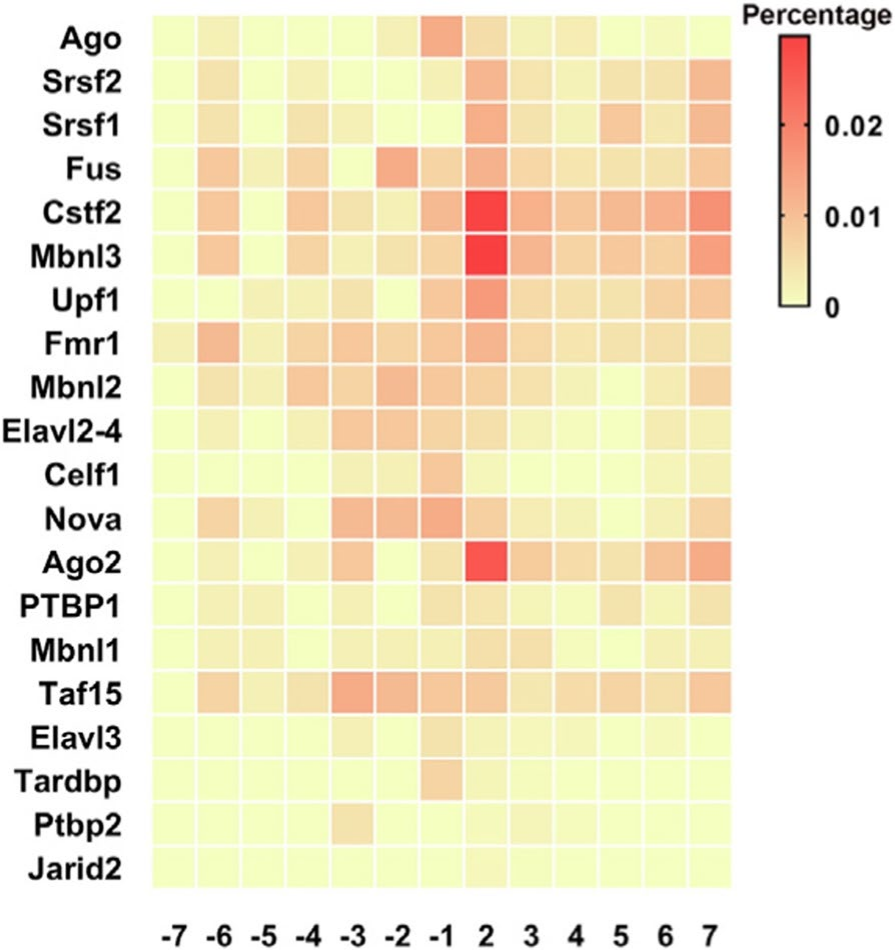
Analysis of RNA-binding protein data. All altered methylated m^6^A peaks (fold change > 2 and *p* < 0.05) were divided into several groups according to the log2 (fold change) values. Based on the value of RNA-binding protein in different groups, the heatmap was used to show the distribution

To exert the biological function of m^6^A modifications, RBPs that recognize specific sites are necessary. In this study, the RMBase v2.0 database was used to identify potential RBPs of differentially methylated m^6^A sites. Relative information about m^6^A modification sites and the support list of RBPs was obtained from this database. In total, we identified 20 potential RBPs in the upmethylated peaks and 19 (missing Jarid2) potential RBPs in the downmethylated peaks. The RBPs were highly distributed in the differentially methylated m^6^A sites with a fold change (log2) of approximately 2. According to the percentage of RBP-bound m^6^A sites in the differentially methylated m^6^A sites, this distribution is presented as a heatmap in Fig. 7, which shows that Cstf2, Ago2, and Mbnl3 are widely distributed.

### Conjoint analysis of m^6^A-RIP-seq and RNA-seq data

**Fig. 8:**
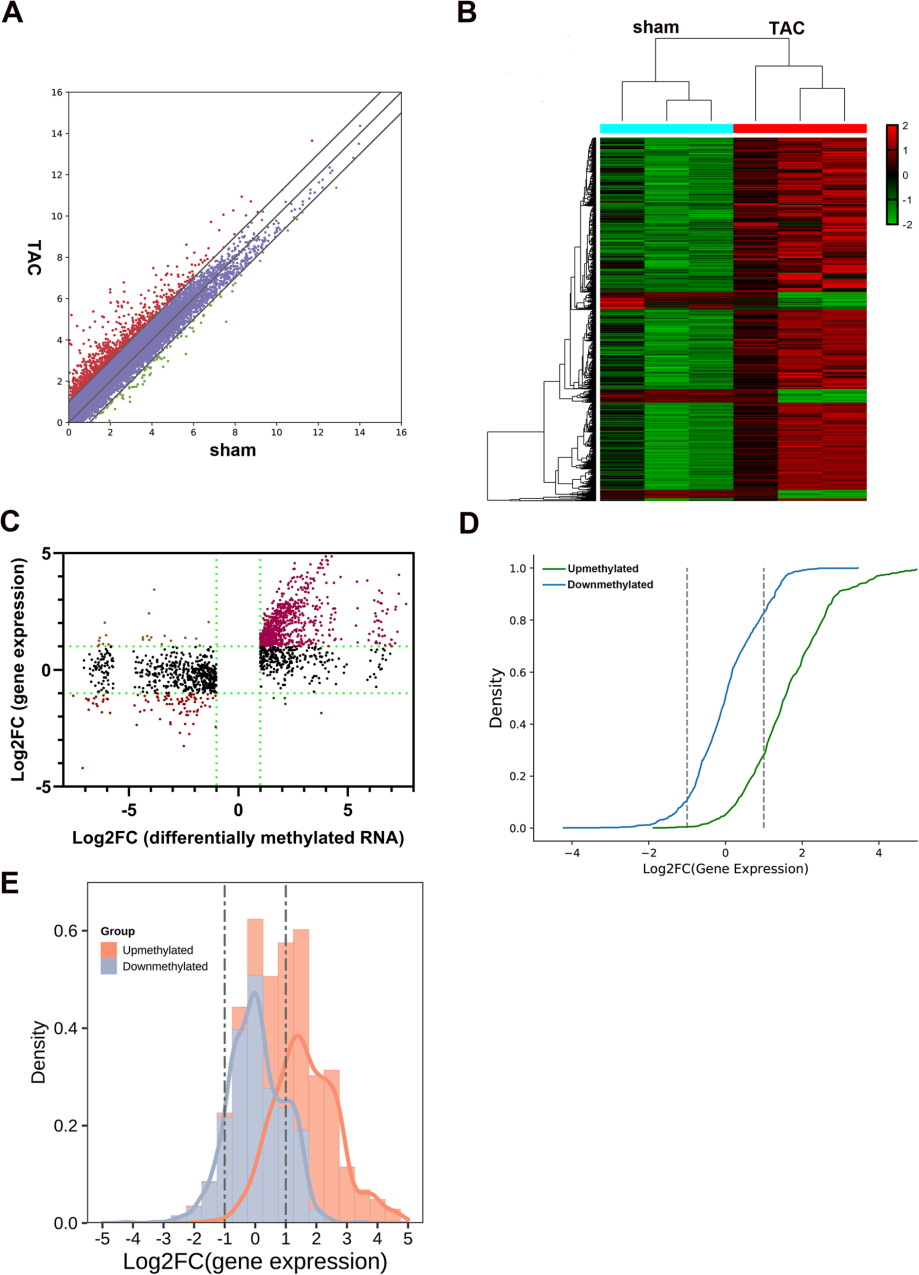
Conjoint analysis of MeRIP-seq and RNA-seq data from hypertrophic heart tissues in mice. **A** Differentially expressed mRNAs between sham and transverse aortic constriction groups (TAC) (fold changes > 2 and *p* < 0.05); **B** Distribution pattern of altered genes; **C** Distribution of transcripts with a significant change in both m^6^A level and expression after TAC, genes with Log2 fold change > 1 were divided by using green dashed lines; **D** Cumulative distribution function plot showing the correlation between the genes with differentially methylated and expression; **E** Histogram and probability density function plot showing the distribution of two groups base on gene expression

RNA-seq was used to obtain the data of differentially expressed genes in hypertrophic mice hearts. Compared with the control group, 1281 significantly higher genes and 154 significantly lower genes (fold change > 2 and *p* < 0.05) were identified in the TAC group (Fig. 8A). Hierarchical clustering was performed to identify distinguishable gene expression patterns between TAC and sham group (Fig. 8B). Based on the conjoint analysis of RNA-seq and MeRIP-seq data, we found that the upmethylated protein-coding genes were more likely to have higher transcription expression (70.6% with Log2FC (gene expression) > 1) and the downmethylated protein-coding genes were more likely to have lower transcription expression (13.8% with Log2FC (gene expression) < -1) (Fig. 8C-E). The effect of m^6^A modification on genes is mainly achieved by m^6^A reader proteins, which suggests that when cardiac hypertrophy occurs, changes in the level of m^6^A modification on mRNA are likely to affect the binding of stability-related reader proteins.

### M^6^A modification enhancing KCNN2 and BMP4 mRNA stability in cardiac hypertrophy

**Fig. 9:**
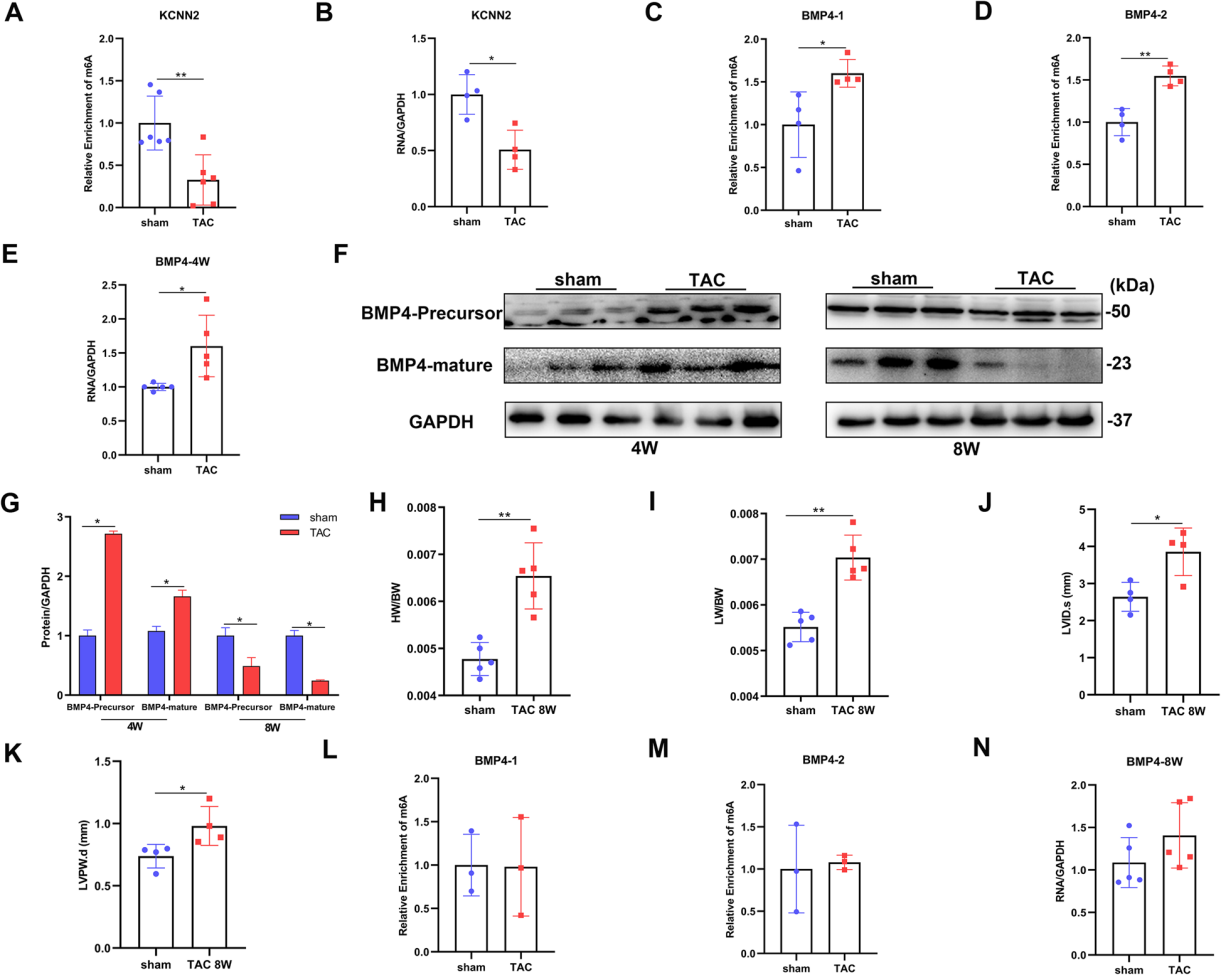
The correlation between m^6^A modification and mRNA expression of KCNN2 and BMP4 in hypertrophic mice hearts. **A** MeRIP-qPCR analysis of the m^6^A modification on KCNN2 mRNA after four weeks of TAC (*n* = 6 each); **B** qRT-PCR analysis mRNA expression level of KCNN2 after four weeks of TAC (*n* = 4 each); **C**-**D** MeRIP-qPCR analysis of the m^6^A modification on BMP4 mRNA after four weeks of TAC (*n* = 4 each); **E** qRT-PCR analysis mRNA expression level of BMP4 after four weeks of TAC (*n* = 5 each); **F** Representative immunoblots of BMP4; **G** Densitometry quantification of protein (*n* = 3 ~ 5 each); **H-I** Gravimetric analysis of heart weight normalized to body weight (HW/BW) and lung weight normalized to body weight (LW/BW) after eight weeks of TAC (*n* = 6 each); **J**-**K** Measurements of internal dimension at end-systolic (LVID.s) and left ventricular posterior wall thickness at end-diastole (LVPW.d) after eight weeks of TAC (*n* = 4 each); **L**-**M** MeRIP-qPCR analysis of the m.^6^A modification on BMP4 mRNA after eight weeks of TAC (*n* = 3 each); **N:** qRT-PCR analysis mRNA expression level of BMP4 after eight weeks of TAC (*n* = 5 each). The results are expressed as the means ± SD (*p < 0.05, compared to the sham group)

KCNN2 is a voltage-independent K^+^ channel activated by intracellular Ca^2+^, expressed in almost all excitable cells, and BMP4 is a member of the bone morphogenetic protein family [25, 26]. Both KCNN2 and BMP4 have been confirmed to play essential roles in cardiac hypertrophy. The abnormal expression of KCNN2 after cardiac hypertrophy may be related to some important heart process [27]. BMP4 act as a pro-hypertrophic factor in the heart. Many studies have shown that BMP4 is related to the development of the heart and mediated cardiomyocyte hypertrophy and myocardial fibrosis [28, 29]. To verify the effect of m^6^A methylation modification on gene expression, we detected m^6^A modification level and mRNA expression level of KCNN2 and BMP4 after TAC (relevant information was obtained from MeRIP-seq and RNA-seq). Based on MeRIP-qPCR and qRT-PCR analysis, the m^6^A modification level and mRNA expression level of KCNN2 were significantly decreased compared with the control group after four weeks of TAC (Fig. 9A and B). Moreover, there are two methylated m^6^A sites on BMP4 mRNA which were significantly increased compared with the control group (Fig. 9C and D), and the mRNA expression level of BMP4 was also significantly increased compared with the control group (Fig. 9E).

Notably, the immunoblots results showed that the precursor and mature of BMP4 protein were significantly increased at four weeks post-TAC, but markedly decreased at eight weeks post-TAC (Fig. 9F and G). So we further detected the m^6^A modification level and mRNA expression level of BMP4 at eight weeks post-TAC to verify that the changes in protein expression level were caused by m^6^A modification. The results of M-mode echocardiograms showed that after eight weeks of TAC, HW/BW, LW/BW and left ventricular posterior wall thickness were significantly increased in TAC group, and the mice developed myocardial hypertrophy (Fig. 9H-K). Subsequently, we found that the upregulated of the two methylated m^6^A sites on BMP4 mRNA measured at four weeks post-TAC disappeared (Fig. 9L and M), and the mRNA expression levels of BMP4 was not significantly changed compared with the control group (Fig. 9N). Besides, the KCNN2 and BMP4 mRNA expression levels in this study were consistent with the results of RNA-seq (GSE182985) obtained from GEO database. Taken together, these results indicate that m^6^A modification may promotes KCNN2 and BMP4 mRNA stability when mice develop cardiac hypertrophy.

## Discussion

In this study, TAC was performed to cause pressure overload in mice hearts to induce cardiac hypertrophy. We observed that the cardiac tissue responds to changes in the environment, such as increased cardiomyocyte volume in the face of mechanical overload, to compensate for variation in cardiac function, thereby maintaining normal cardiac function despite hypertrophy. Studies have confirmed that m^6^A modifications in the body also respond to changes in the environment and time. For example, when neonatal cardiomyocytes develop until the seventh day, the m^6^A modification level decreases, which may be related to the downregulation of *METTL3* expression [30]. Additionally, altered m^6^A modifications may affect the proliferative capacity of cardiomyocytes [31].

This study found that cardiac hypertrophy occurred in mice four weeks post TAC and the level of m^6^A modification of total RNA increased in hypertrophic heart tissues, which may be related to changes in the expression of these key methyltransferases or demethylases. The results of qRT-PCR and western blotting analyses showed that the gene and protein expressions of *FTO* and *WTAP* were significantly downregulated, whereas the mRNA level of *METTL3* showed no significant change, but there was an increase in the protein expression. However, it is unclear whether the altered level of m^6^A modification after hypertrophy is mediated by a single enzyme or by the synergy of multiple enzymes; this requires further studying. However, the key methyltransferase *METTL3* may play an important role in cardiac hypertrophy, and studies have found that *METTL3* may be a key regulatory factor in increasing m^6^A methylation levels after hypertrophy. The inhibition of *METTL3* in mice hearts can lead to heart failure, whereas the overexpression of *METTL3* promotes the development of hypertrophy in cardiomyocytes, highlighting the critical importance of m^6^A RNA methylation in the heart for maintaining normal cardiac function [32]. In addition, we focused on the profiles of altered m^6^A modified transcripts and gene expression after hypertrophy, and biological function analysis of these changed genes was performed.

Analysis of the distribution profiles of m^6^A peaks within mRNAs in heart tissues revealed that the m^6^A sites are typically harbored in CDS and stop codon segments [33]. Furthermore, the results of the preferential location of m^6^A modifications showed that the main differences between the two groups were concentrated in the CDS region. This was also confirmed by IGV alignment of genes with differential m^6^A modifications in the MeRIP-seq data, suggesting that the effect of m^6^A modifications on cardiac hypertrophy may be related to its effects, such as altering translation and mRNA splicing, on the CDS region of the mRNA [34, 35].

GO and KEGG pathway analyses of differentially methylated genes showed a strong correlation with heart function, such as heart process and heart contraction (ontology: biological process), hypertrophic cardiomyopathy, and dilated cardiomyopathy (pathway analysis). We also used the RMBase v2.0 database to identify potential RBPs of differentially methylated m^6^A sites, and the results showed that Cstf2, Ago2, and Mbnl3 were widely distributed, which may play a critical role in RNA splicing through RNA binding [36].

Finally, based on a conjoint analysis of MeRIP-seq and RNA-seq data, we found that the higher the methylation ratio, the higher the transcription expression, which was consistent with studies in patients with high myopia [37]. In this study, we found the m^6^A modification level of BMP4 and KCNN2 were consistent with their gene expression. This suggests that changes in the level of m^6^A modification of genes are likely to affect gene expression [38]. At present, many studies have confirmed that YT521-B homology (YTH) and insulin-like growth factor 2 mRNA-binding protein (IGF2BP) domain play a role in mRNA stabilization, translation and degradation by recognizing methylated m^6^A sites on genes [39–41]. The conjoint analysis of MeRIP-seq and RNA-seq data in this study suggest that when the level of m^6^A modification increases, the expression of the gene may increase by promoting the stability of the gene. One of the features of cardiac hypertrophy is increased protein synthesis, which suggests that m^6^A modification may play an important role in cardiac hypertrophy through increases the expression of related proteins genes by stabilizes target mRNA [42]. Furthermore, previous studies have shown m^6^A modification promoted YTHDF2 protein expression through enhancing Ythdf2 mRNA stability in cardiac hypertrophy [43]. In addition, many genes with changes in both m^6^A modification and gene expression are related to cardiac processes, such as *Myh7, bmp4* and *Ttn*, which were shown to be related to hypertrophic cardiomyopathy [44–47]. These results may provide guidance for future studies on the role of m^6^A methylation in cardiac hypertrophy.

## Conclusion

In conclusion, we performed m^6^A-RIP-seq and RNA-seq to analyze the transcriptome profiles of genes with altered m^6^A modification and expression, revealing the potential functions of altered transcripts and RBPs, thereby providing a fundamental contribution to future studies on cardiac hypertrophy.

## Supplementary Information

**Additional file 1: Supplemental figure 1. **Full length of representative immunoblots.

## Data Availability

The authors confirm that the data supporting the findings of this study are available within the article. The m^6^A-sequencing and RNA-sequencing datasets have been submitted to the GEO database under the accession number GSE201764.
