# Supplementary material for: Comprehensive analysis of RNA m6A methylation in pressure overload-induced cardiac hypertrophy
Source: BMC Genomics. 2022 Aug 11;23:576. doi: 10.1186/s12864-022-08833-w (PMC9373449; doi:10.1186/s12864-022-08833-w)
Supplement: Supplementary file 1 — Additional file 1: Supplemental figure 1. Full length of representative immunoblots. [file 12864_2022_8833_MOESM1_ESM.docx]

**Supplemental figure 1. Full length of representative immunoblots.**


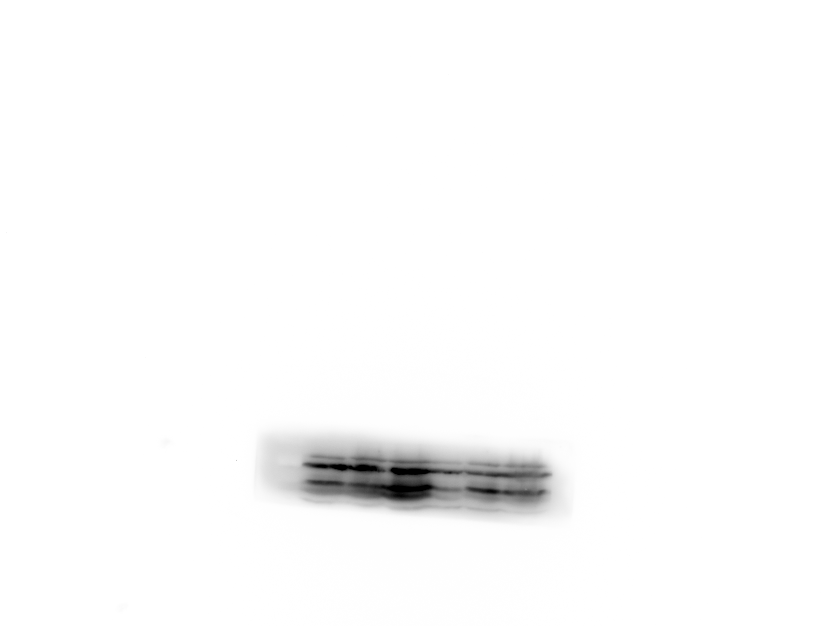


METTL3


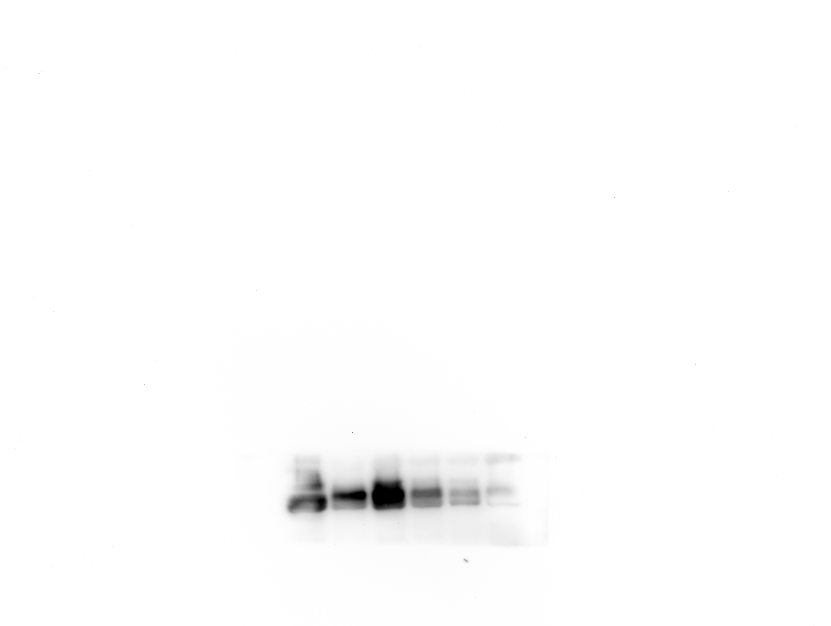


FTO


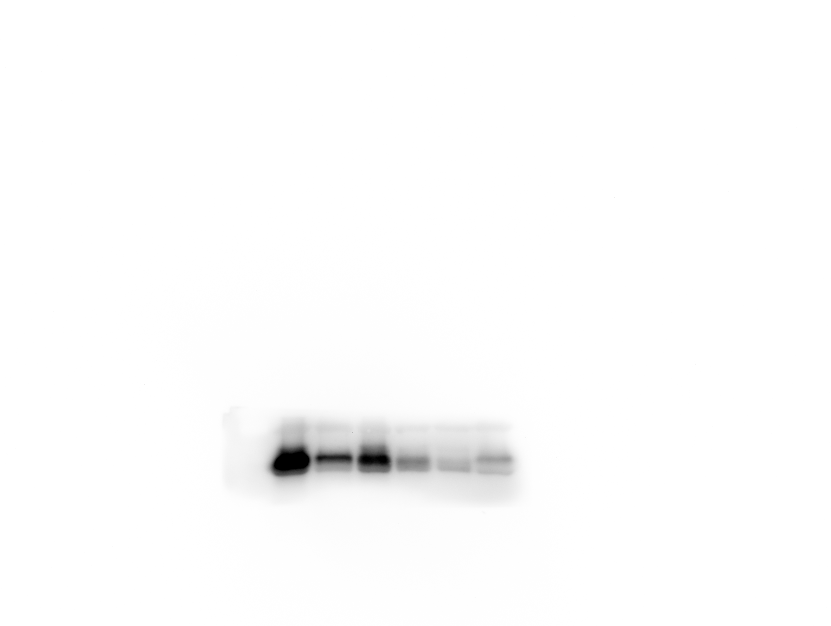


WTAP


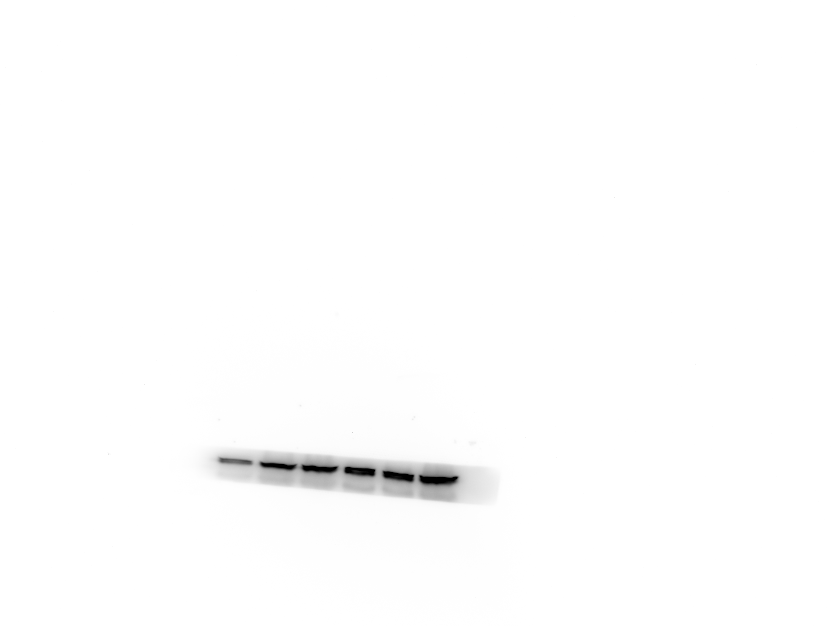


ALKBH5


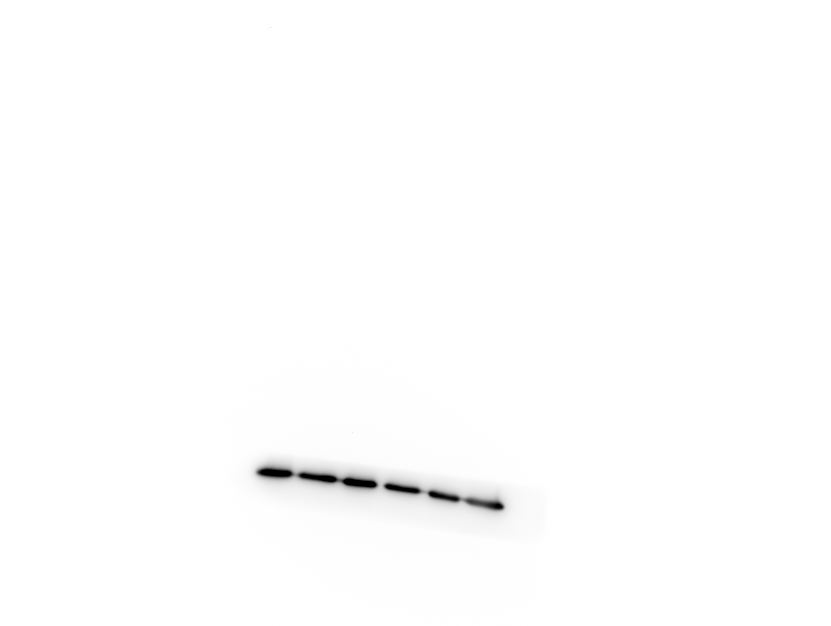


GAPDH


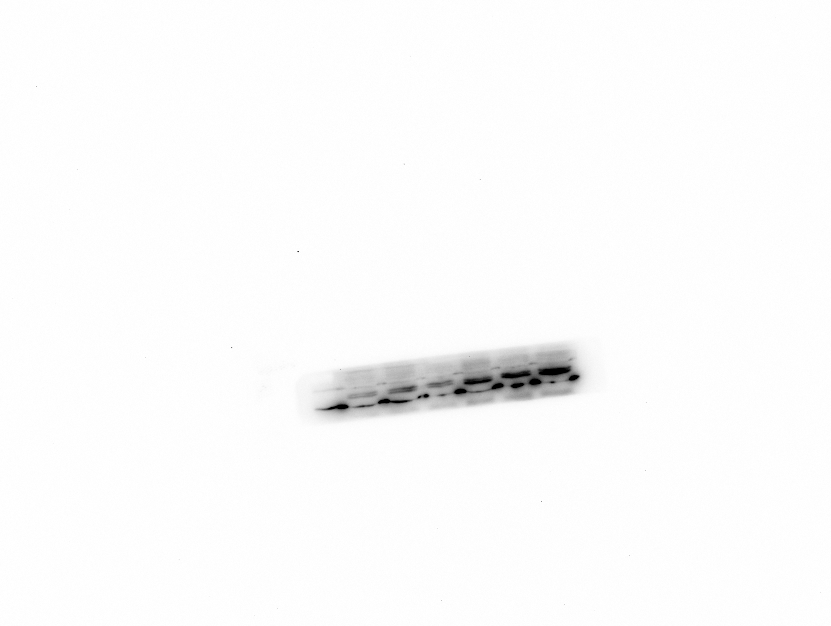


BMP4-Precursor-4W


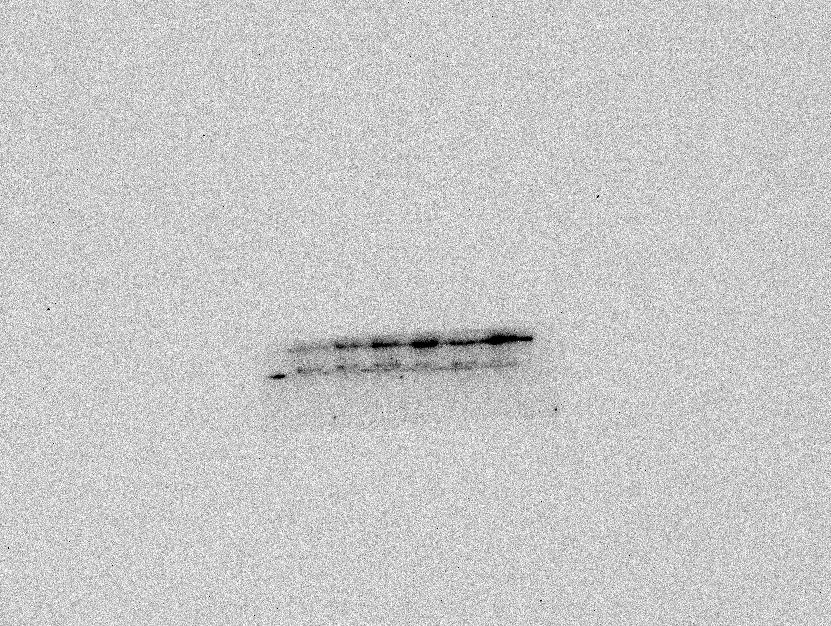


BMP4-mature-4W


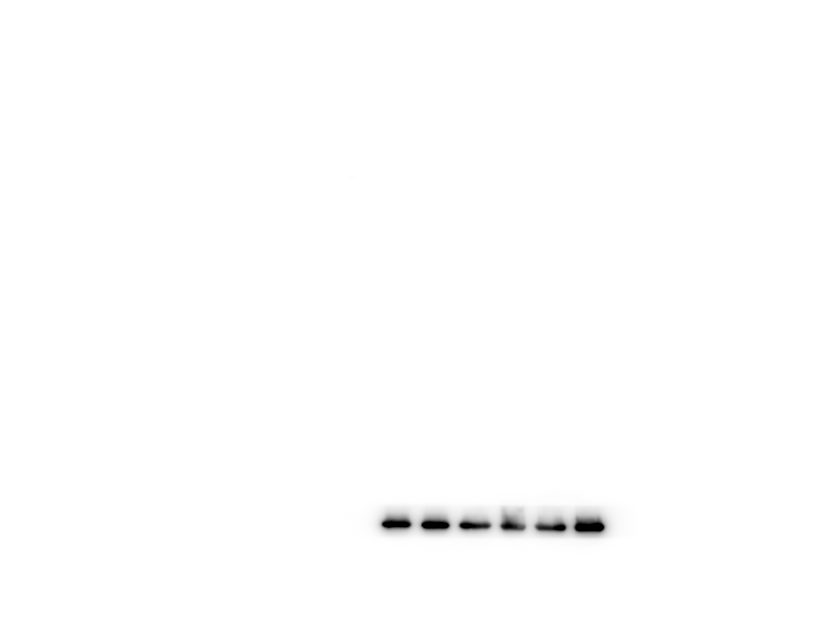


GAPDH-4W


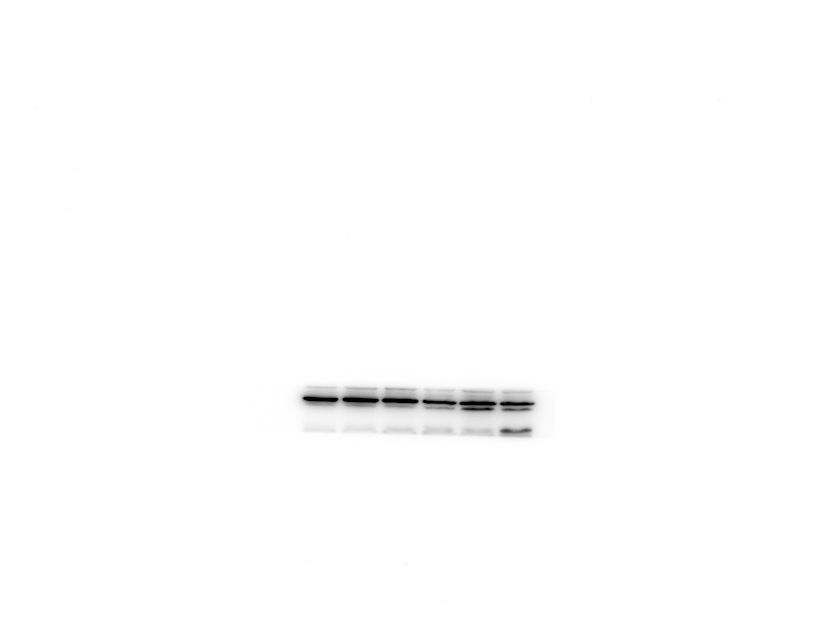


BMP4-Precursor-8W


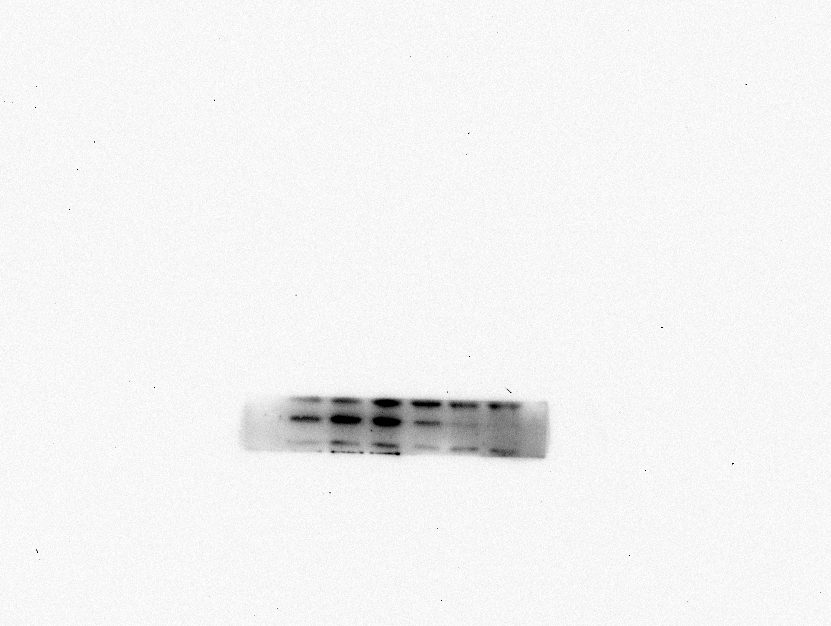


BMP4-mature-8W


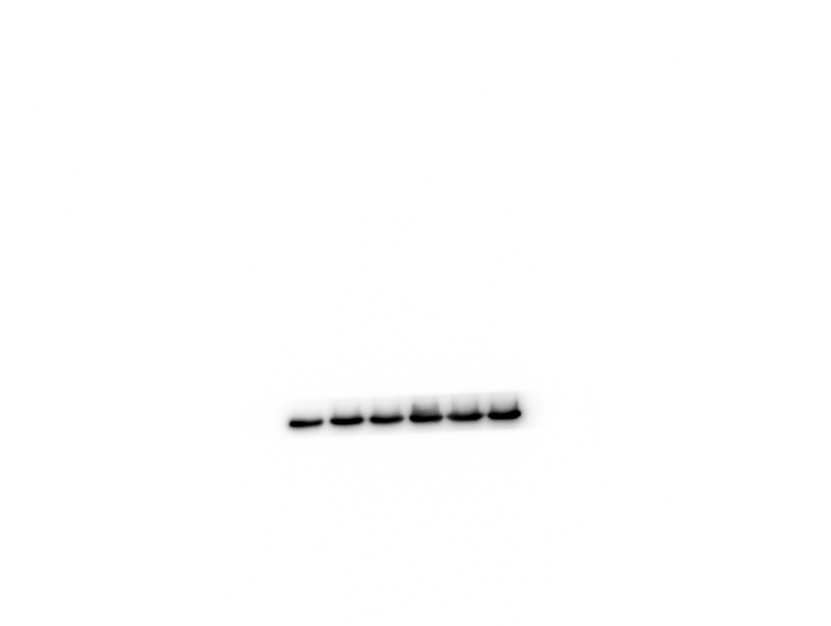


GAPDH-8W
